# 

**Published:** 1925

**Authors:** 


### CONTENTS.

| | Page |
|---|---|
| Continuity and Change. By T. Carwardine, M.S., F.R.C.S. ... | 201 |
| Some Problems in Pulmonary Disease, with Special Reference to Radiography. By H. H. Carleton, M.A., M.D. (Oxon.), M.R.C.P. ... | 211 |
| The Practical Importance of Posture in the Physical Examination of the Heart, with some Remarks on Stethoscopes. By W. Gordon, M.D., F.R.C.P. ... | 224 |
| Reviews of Books ... | 232 |
| Editorial Notes ... | 241 |
| The Library of the Bristol Medico-Chirurgical Society ... | 244 |
| Local Medical Notes ... | 246 |

